# Model-Based Methods in the Biopharmaceutical Process Lifecycle

**DOI:** 10.1007/s11095-017-2308-y

**Published:** 2017-11-22

**Authors:** Paul Kroll, Alexandra Hofer, Sophia Ulonska, Julian Kager, Christoph Herwig

**Affiliations:** 1 Research Area Biochemical Engineering, Institute of Chemical Environmental and Biological Engineering, Vienna University of Technology, Gumpendorfer Straße 1a – 166/4, A-1060 Vienna, Austria; 2 Christian Doppler Laboratory for Mechanistic and Physiological Methods for Improved Bioprocesses, TU Wien, Vienna, Austria

**Keywords:** bioprocess, data mining, modelling, monitoring, optimization

## Abstract

Model-based methods are increasingly used in all areas of biopharmaceutical process technology. They can be applied in the field of experimental design, process characterization, process design, monitoring and control. Benefits of these methods are lower experimental effort, process transparency, clear rationality behind decisions and increased process robustness. The possibility of applying methods adopted from different scientific domains accelerates this trend further. In addition, model-based methods can help to implement regulatory requirements as suggested by recent Quality by Design and validation initiatives. The aim of this review is to give an overview of the state of the art of model-based methods, their applications, further challenges and possible solutions in the biopharmaceutical process life cycle. Today, despite these advantages, the potential of model-based methods is still not fully exhausted in bioprocess technology. This is due to a lack of (i) acceptance of the users, (ii) user-friendly tools provided by existing methods, (iii) implementation in existing process control systems and (iv) clear workflows to set up specific process models. We propose that model-based methods be applied throughout the lifecycle of a biopharmaceutical process, starting with the set-up of a process model, which is used for monitoring and control of process parameters, and ending with continuous and iterative process improvement via data mining techniques.

## Introduction

A safe product is the target of every production process. In the field of pharmaceutical products, this is ensured by elaborate approvals and the continuous control of independent authorities such as the Food and Drug Administration or the European Medicines Agency. The International Conference on Harmonisation (ICH) has established quality guidelines which should be considered during process lifecycle (1). A process lifecycle includes process development, scale up and continuous optimization until product discontinuation. The basis of a production process is a definition of the product by a quality target product profile (QTPP) that includes critical quality attributes (CQA) (2), such as physicochemical properties, biological activity, immunochemical properties, purity and impurities (3). The aim of each industrial production process is to satisfy the predetermined CQAs with a maximum of productivity. According to the ICH Q8(R2) guidelines the quality by design (QbD) approach is a one way of engineering an adequate production process. QbD combines sound science and quality risk management in order to identify critical material attributes (CMA) and critical process parameters (CPP), which shows significant effects on CQAs. In addition, the functional relationships between CMAs and CPPs on CQAs should be investigated (2). This requires the use of mathematical models within the framework of QbD. The basic idea of the QbD approach is that a process with controlled CMAs and CPPs in a defined design space will lead to continuous CQAs and finally to a sufficient QTPP. In order to achieve this goal process analytical technology (PAT) – tools are used. PAT includes the tasks of designing, analyzing and controlling production processes based on real-time monitoring of critical parameters including them CMAs, CPPs and CQAs (2,4).

**Fig. 1:**
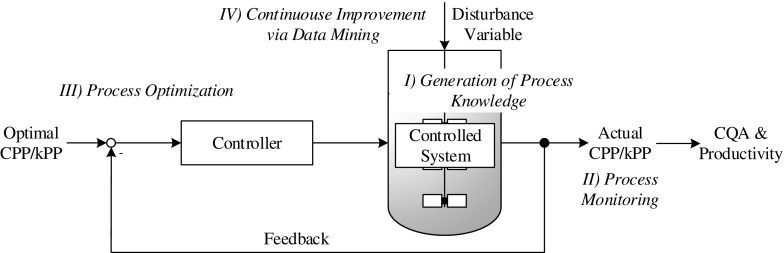
A simple control loop with the related four challenges (I-IV) of process development and the process lifecycle. Challenge I is the generation and storage of knowledge within models. Challenge II is the process monitoring. Challenge III is the determination of optimal process conditions for different applications and IV the continuous improvement of a process by data mining tools.

In addition to adequate product quality, each process aims for high productivity. This includes the thoughtful use of raw materials, technologies and human resources in addition to the reduction of unwanted by-products. In contrast to CPPs which only affect product quality, key process parameters (kPP) affect productivity and economical viability (5). During the whole process lifecycle, CPPs and kPPs have to be improved in order to react to changed boundary conditions such as fluctuations in raw materials, new production facilities and locations, new technologies and constantly fluctuating staff. In summary, the following four challenges arise during a process lifecycle: I) generation of process knowledge, II) process monitoring, III) process optimization and IV) continuous improvement of the process (Fig. 1). In order to fulfill these challenges during the entire process lifecycle, a lifecycle management is indispensable. ICH Q8(R2) and Q12 address this issue but don’t give any concrete solution (2,6). The reason for this is the lack of practicable knowledge management systems (7).

In order to solve the four previously presented challenges during the process lifecycle an overview of available methods and technologies is given in the present manuscript. The focus lies on model-based methods which are characterized by the use of mathematical models. Basically, each process model can be described by an Eq. [1], which is defined by a model output/outputs *y*, a function *f*, the time *t*, model states *x*, and the design vector *φ*, including all necessary process parameters (CPPs & kPPs) such as feed rates, temperature, pH etc., and the model parameters *θ*.

1 $$ y=f\left(t,x,\varphi, \theta \right) $$

In order to show the interaction between the four separate challenges (I-IV), the red line of this paper will be analogous to a simple control loop (Fig. 1). This approach allows a scientific discussion of interaction and a possible outlook with respect to the process lifecycle.

The first challenge (challenge I) investigated is the identification of CPPs, kPPs and the generation of process knowledge. Process development and improvement can only occur if relationships and interactions are understood. With respect to model-based methods process relevant (critical) knowledge is defined as the sum of relationships and interactions, which should be considered in a process model in order to predict a target value (CPP, kPP or CQA). Modelling is a tool for the identification and description of these relationships with mathematical equations verified by statistic tests. In chapter 2.1, different modelling workflows will be presented and compared with respect to the modelling goal, complexity as well as transferability to biopharmaceutical production processes. The basis of the parametrization and verification of each model are data. Especially in model development, data have a major impact on model structure and validity space. Therefore, there is a strong iteration between modelling and data collection. In the second part of chapter 2.1 methods are described as to which data should be collected to verify the process model. The main output of this chapter are methodologies in order to generate adequate model structures *f* and model parameters *θ*, which can be adapted during the process lifecycle based on additional data and knowledge. The models and their parameters are necessary for further monitoring and control applications.

Since every process is affected by certain disturbances which affect quality and productivity, monitoring is a need for biopharmaceutical production processes (challenge II) (2). Monitoring is defined as the supervision of process parameters and variables, which is needed for subsequent control actions. Monitoring hereby includes the collection of information by measurements and subsequent data processing, whereas in the latter model-based methods can be applied. These methods and their application in monitoring will be discussed in chapter 2.2. It will focus on methods which help to define the needed measurements, allow the combination of multiple measurements to handle process noise and measurement uncertainty and finally allow the estimation of unmeasured states.

The final aim of process design is process control, discussed in chapter 2.3. Mainly two topics will be discussed within the challenge III) “Process Optimization”. The first topic is a clear description of the control goal within certain boundaries that are based on product, technical, physiological and economic limits. Thereby various model-based methods for open-loop and closed-loop applications will be presented. With regard to model-based methods, methodologies for optimal and predictive control are presented. The second topic is estimating an “optimal” design vector and identifying critical process limitations, which provide an important input for further process optimization.

Certain disturbances that affect every process can be classified as a) known but neglected and b) unknown and neglected ones. Both can have a significant impact on process performance and should be continuously improved. This continuous improvement (challenge IV) is a key innovation motor for existing processes during their entire lifecycle. New analytical methods, measurement devices, automation, further data evaluation and others can lead to process relevant knowledge which should be taken into account. Within chapter 2.4 this continuous improvement of the process will be investigated. Regarding model-based methods the focus will be on data-mining tools, which allow researchers to set up hypotheses of potential correlations. These hypotheses are a necessary input for further process model-extensions and support the overall goal of an adequate product quality and high productivity throughout the entire process lifecycle.

Finally, an overall statement on further applications and perspectives of model-based methods within the biopharmaceutical process lifecycle is presented in the conclusion.

## Results & Discussion

### Generation of Process Knowledge

#### Modelling

Within the process lifecycle, knowledge is defined as the ability to describe relationships between (critical) process parameters and critical quality or performance attributes. This knowledge needs to be documented. The trend of the last years is clearly from a transfer approach, which is based on spoken and written words, towards a model approach (8). In the context of biopharmaceutical processes, this indicates the possible usage of process models as knowledge storage systems (9). The setup of these process models is still challenging. Contributions presenting workflows for modeling are increasing (10–15). According to good modelling practice, the single steps of modelling are always similar (14). These steps are: i) setup of a modelling project, ii) setup of a model, iii) analysis of the model. In addition, the documentation of the complete modelling project should be entire and transparent.

The basis of each modelling workflow is a clear definition of the model goal. This often resents a major challenge and cannot be achieved without iterations between modelers and project managers. The model goal should include the definition of target values, acceptance criteria and boundary conditions. Furthermore, the application of the model should be considered. Each process related model should be as simple as possible and as accurate as necessary. From this dogma, it follows that a model should only include necessary (critical) states, model parameters and process parameters. Depending on the goal of the model, different model types are suitable. Frequently used is the classification between data driven, mechanistic and hybrid models (16). In terms of applications of models, the classification between dynamic and static models is more appropriate. Dynamic models include differential equations, typically over time or location coordinates which allow prediction. Static models are correlations which cannot provide time-dependent simulation results. Hence, they are not applicable for prediction over time or location, which is commonly required in bioprocess development. Data driven, mechanistic as well as hybrid models can be both, static and dynamic.

**Fig. 2:**
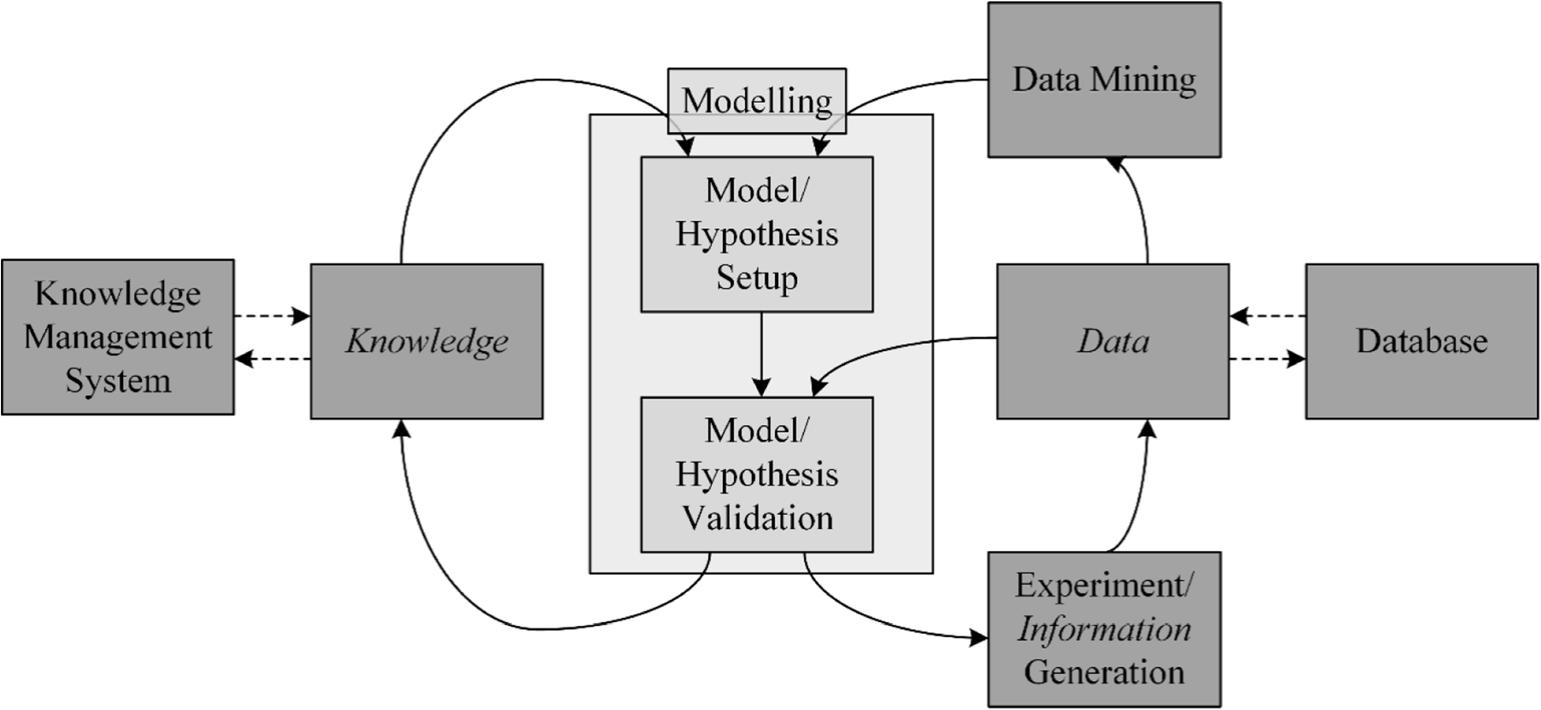
Systematic overview of a model-development including interlinks between data, database and datamining, information and necessary experiments and knowledge.

The set up and analysis of a model are iterative steps within each modelling workflow (13,17), which are illustrated in Fig. 2. For the setup of models, different approaches are reported in literature. To date, experts are required to set up models, as they strongly depend on prior knowledge. This limited prior knowledge is a general gap for the application of all model-based methods. Only a few workflows for automated modelling are available. With regard to the process lifecycle, the focus of this review will be on these automated workflows for the setup of dynamic mechanistic models. A generic and strongly knowledge-driven approach is shown by the company Bayer AG (18,19): Based on an extensive dynamic metabolic flux model in combination with a generic algorithm, the initial complex model is reduced to the most necessary parts. The benefit of this top-down approach is the intense use of prior knowledge. The working group of King shows another approach, based on the detection of process events in combination with a model library (10,20,21). The benefit of this approach is that less prior knowledge is necessary and the transferability on other bioprocesses is given. As one of the drawbacks of model-based methods is the validation of models and there parameters an automated workflow for the generation of substantial target-oriented mechanistic process models was developed in our working group (22). This approach allows the generation and validation of process models with less prior knowledge and without model libraries.

**Fig. 3:**
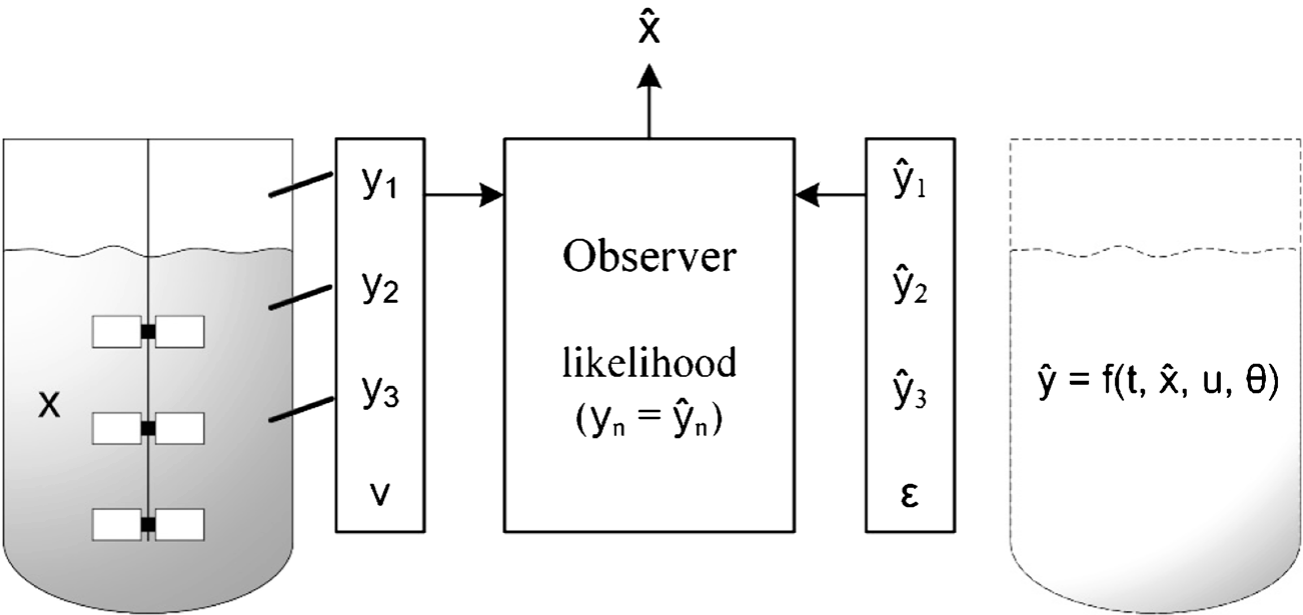
Principle of model based monitoring with multiple measurements. Through the reconciliation of measured model outputs with current model simulations actual process states can be estimated by considering measurement and process uncertainty.

**Fig. 4:**
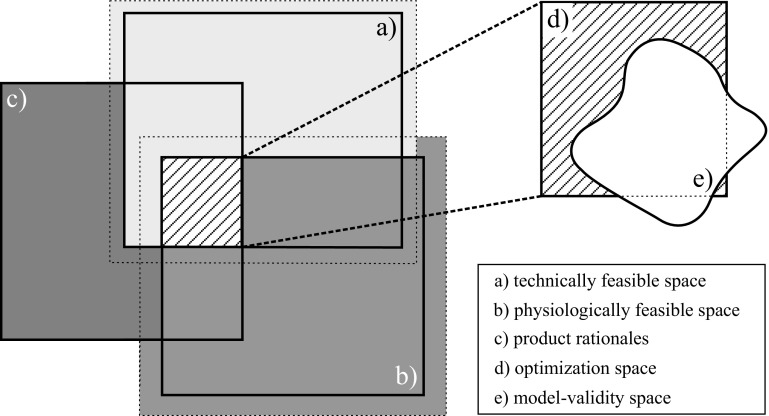
Optimization space limited by technically and physiologically feasible space as well as by product and system rationales. The potential innovation space is the space where it can be increased e.g. by more knowledge about the system.

**Fig. 5:**
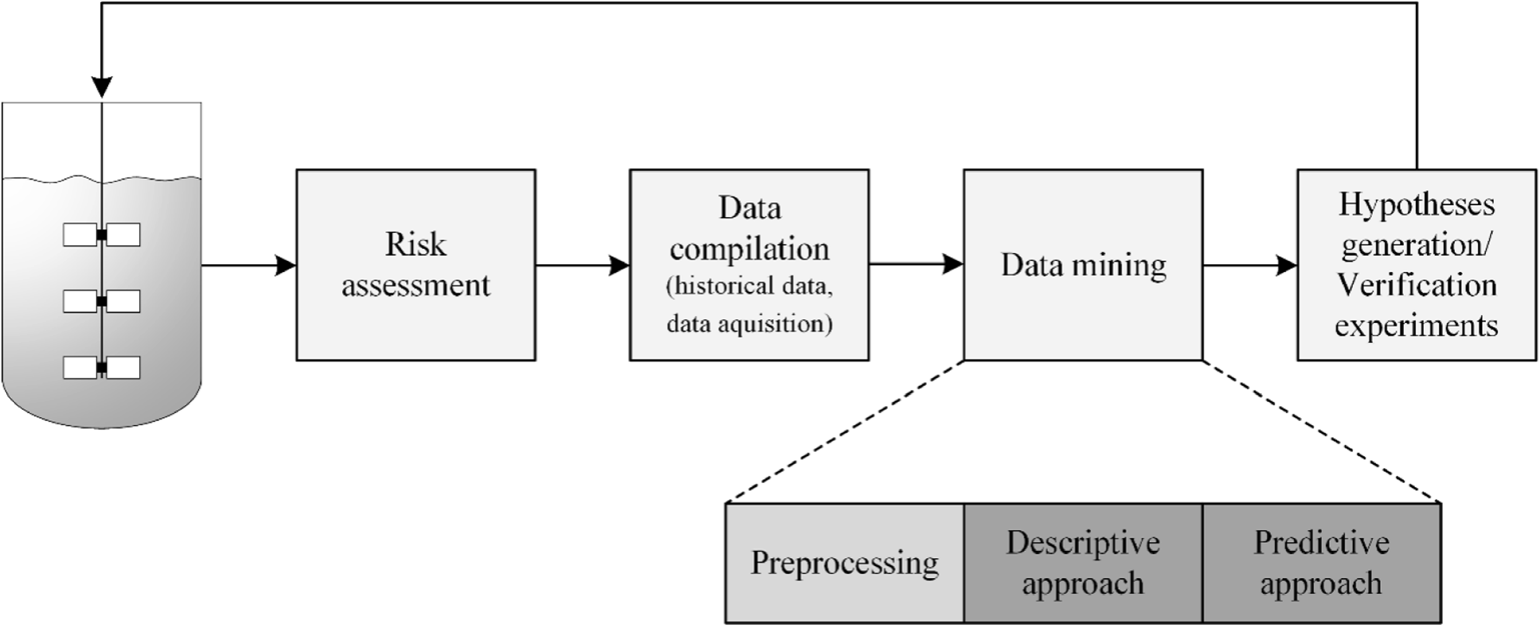
Workflow showing the data-driven knowledge discovery approach for the detection and minimization of disturbance variables. After selection of the targeted disturbance class via risk assessment tools, data has to be generated and/or accumulated. Indications about disturbing variables/ descriptors can be generated by correlation analysis or – if possible – via mechanistic modelling. Obtained knowledge/ information has to be implemented in the design space to allow minimization of the identified disturbances.

The analysis of each model follows the same order. Based on collected data and an assumed model structure a parameter fit is performed. With the use of optimization algorithms, the model parameters are adapted in a way, that the previous defined descriptor is optimized (see chapter 2.3). Typically, this is a minimization of a model deviation, which can be described by different characteristics such as the sum of square errors (SSE), a normalized root mean square error (NRMSE), a profile likelihood or other descriptors. A comparison of the achieved descriptor with a previously defined acceptance criterion is the first analysis of each model. If this fails, the model structure is not suitable for the present issue. If the model passes, the model structure could be suitable to describe the relation. The next analysis is focused on the model parameters $\widehat{\theta}$ and their deviations. Therefore, typically, an identifiability analysis is performed which follows two aims: The first aim is the structural identifiability of model parameters, which is necessary for process models with the aim of monitoring and control. If structural identifiability is not given, model parameters can compensate each other due to cross correlation. This results in multiple solutions and can lead to spurious results. There are several methods in order to evaluate structural identifiability (23,24). If structural identifiability is given, practical identifiability should be investigated in order to fulfil the second goal, which is a statement about confidence intervals of model parameters based on existing data (24,25). This is necessary in order to decide if parameters can be estimated with the data available. If practical identifiability is given, the model parameters are significant. If not, two statements can be made: i) the available data allows no determination of the model parameter or ii) the model structure allows cross correlations between model parameters and is therefore not as simple as possible.

In addition to the analysis of model and model parameter deviations, there is a variety of methods to characterize models with their focus on robustness. The first check should be a global behavior test with the goal of ensuring the right implementation of a model: here the model is tested with extreme input values. Additionally, if possible, certain redundancy should be implemented in the model (see chapter 2.2). Typical approaches are material balances, as they are typically used for yeast or microbial processes (13,26). Another frequently used method is a sensitivity analysis with the aim of showing the impact of deviations of model parameters, process parameters and model inputs on model outputs (27,28). The information obtained in this sensitivity analysis can be used to improve the model within the process lifecycle. With respect to the further usage of models certain causes for deviations must be considered. Deviations can be mainly obtained from two sources. The first source of deviations is the model structure in itself. Based on the principle that a model is always a sum of assumptions, there is always an accepted model deviation with a predefined validity space. In addition, models can always only explain a part of the process variance. Disturbances that are not considered in the model cannot be explained by it. Within the concept of process lifecycle this implies a continuous model improvement (see chapter 2.4). The second source of variance is the deviation of the model parameters $\widehat{\theta}$ caused by changing and not explained sources of variance. This can be improved by adapting the model structure or parameters. For both additional information is necessary. It can be provided by additional data or additional hypotheses from data mining methodologies (see chapter 2.4) leading to new model structures (Fig. 2). With respect to real-time application, several methodologies for model adaption are shown in chapter 2.2.

#### Generation of Information

During the process development certain experiments must be performed in order to identify CPPs and an adequate design space and to verify process models. The most widely used strategy is the standard design of experiments (DoE) (29), which is given as an example in the guidelines ICH Q8(R2) (2). However, the applicability of standard DoEs for bioprocesses comprising a huge number of potential CPPs is not given to the full extent. The reason for this is mainly the model-based data evaluation, which typically only assumes linear or quadratic effects between process parameters and quality/product attributes. Known relationships describing physiological interactions are usually not taken into account in standard DoEs. Therefore other model-based methods are available which are based on information.

In order to verify certain process models, information is necessary. Within this context, information is defined as the possibility of estimating the model parameters *θ* of a model *f* with collected data. Mathematical statistics call this the Fisher information, which is described by the Fisher information matrix (*H*_*θ*_). *H*_*θ*_ depends, besides the static model structure, on the model parameters *θ* and the design vector *φ* and can be estimated by Eq. [2] (30,31). The design vector includes all possible process parameters, which are considered in the model, and sampling points (t_k_) where additional data are collected. ${H}_{\theta}^0$ describes the initial fisher information matrix, n_sp_ the number of sampling points, *N*_*y*_ the number of model states y and *N*_*θ*_ the number of model parameters *θ*.

2 $$ {H}_{\theta}\left(\theta, \varphi \right)={H}_{\theta}^0+\sum \limits_{k=1}^{n_{sp}}\sum \limits_{i=1}^{N_y}\sum \limits_{j=1}^{N_y}{s}_{ij}{\left[\frac{\partial {\widehat{y}}_i{\left({t}_k\right)}^T}{\partial {\theta}_l}\frac{\partial {\widehat{y}}_j\left({t}_k\right)}{\partial {\theta}_m}\right]}_{l,m=1\dots {N}_{\theta }} $$

Applications for *H*_*θ*_ are mainly found in the model-based design of experiments (MB-DoE). An experiment has per definition the aim to prove, refute or confirm a hypothesis. In the case of MB-DoE the hypothesis is the process model in itself. Therefore, the information content of a planned experiment is maximized depending on *φ*. This information content should be a criterion extracted from *H*_*θ*_. The D-optimal design which indicates a maximization of the determinant of *H*_*θ*_ is frequently used. Other descriptors include the maximization of the trace of *H*_*θ*_ (A-optimal) or the maximization of the smallest eigenvalue (E-optimal) (32). Telen *et al*. investigated additional criteria and showed the applicability of MB-DoE in order to estimate model parameters from a simulated fed-batch study (33). In addition, drawbacks of the single criteria are discussed and a novel multi-objective approach is investigated. This implies that MB-DoE strongly depends on the chosen information criteria. This must be transparent in order to ensure systematic and sound decisions. Table I shows some applications of MB-DoE and a summary of novel approaches to design criteria.

**Table I:** Summary of Applications and Novel Publications with Respect to Model-Based Experimental Design

Method	Criteria	Application	Real-time	Reference
Application paper				
Signal to noise ratio	SNR = const	estimation of sampling points with respect to deviations on specific rates	at-line/ off-line	(34)
Sequential experimental design	D-criteria	experimental design within a model discrimination workflow	at-line/ off-line	(35,36)
Optimal dynamic experiments	–	MB-DoE in microbioreactor systems under use of FTIR spectroscopy as monitoring tool	at-line/ real-time	(37)
Simultaneous solution Approach for MB-DoE	A, D & E - criteria	design of feed rates and adaptive optimal sampling strategy	at-line/ off-line	(38)
CMB-DoE	A, D & E - criteria	adaption of a dynamic experiment under usage of real-time data control on information criteria	real-time	(39,40)
Online optimal experimental re-design	A-criteria	adaption of a dynamic experiment under usage of real-time data control on information criteria	real-time	(41,42)
Model discriminating experimental design	–	Model descrimination within an sequential workflow	at-line/ real-time	(43)
Design criteria paper				
D-optimal design DMOO design (multi objective optimization)		reduction of parameter interactions with MB-DoE under usage of a multi objective optimization criteria	at-line/ off-line	(44)
Multi objective approach		Multi-objective MB-DoE to descriminate between models and estimate kinetic parameters	at-line/ off-line	(45)
Anticorrelation criteria		anticorrelation criteria to estimate model parameters	at-line/ off-line	(46)

However, information is strongly coupled with the identifiability analysis of modelling workflows (see chapter 2.1.1) (44,47–49). As described there, available data are necessary in order to estimate identifiable parameters. MB-DoE is the model-based method solving this issue. Several publications show the application of MB-DoE in order to reduce the experimental effort with the goal of verifying process models. An issue for MB-DoE is the handling with uncertainties based on model and experimental deviations (50). One possibility is the real-time adaption of the experimental design, which is called continuous model-based experimental design (CMB-DoE) (39,40) or online optimal experimental re-design (41,42). This is finally a control issue and strongly related to process monitoring (see chapter 2.2) and optimization (see chapter 2.4).

### Process Monitoring

Process monitoring is the description of the actual state of the process system in order to detect deflections of CPPs or key process parameters in time. With regard to the definition of PAT, monitoring without a feedback for process control is only measurement (51). Process monitoring can be seen in the context of measurement, monitoring, modeling and control (M^3^C) (52). After describing the first tasks of monitoring and the real-time data collection, model based methods for the subsequent data processing are presented, followed by the description of implemented examples in the field of biotechnology, which are also collected in Table II.

**Table II:** Monitoring Solutions within Biotechnology

Monitoring goal	Model scenario	Measurement scenario	Process system	Algorithm	Highlights	Ref.
Biomass growth	mass-balance with fixed stoichiometry	carbon in and outflow	*P. chrysogenum*	SQP (sequential quadratic programming)		(64)
Biomass growth	mass-balance with variable stoichiometry	carbon and electron in and outflow	*P. pastoris* and *E. coli*	–	use of system redundancy	(65)
Oxygen consumption	mass balance	offgas	*CHO*	–	simple and robust	(66)
CO2 production	mass balance	offgas	*CHO*	–	carbonate buffered media	(67,68)
Biomass concentration	kinetic model	sugar measurements	*Daucus carota*	extended kalman filter	field of plant cells	(69)
Substrates & biomass	kinetic model	CO2, sugars, product	*S. cerevisiae*	extended kalman filter	NIR based online measurement	(70)
Product & biomass	kinetic flux model	offgas analysis, product	*P. chrysogenum*	particle filter	Raman based online measurements	(58)
Biomass growth	kinetic model	offline & online offgas	*S. clavuligerus*	extended kalman filter	account for measurement delay	(71)
Biomass growth	kinetic model with energy balance	calorimetry	*E. coli*	–	robust growth determination	(72)

Measurements are a central part of monitoring as they provide the time resolved raw information of the ongoing process. Measurement methodologies and devices should be simple, robust and as accurate as necessary. Besides well-established measurements such as pH, dissolved oxygen and gas analysis, a vast amount of process analyzers is available nowadays; however the development of measurement techniques is still a field for extensive research in Biotechnology (53,54). These include - but are not limited to - chemical /biological measurements, which are characterized by a high sensitivity and by physical sensors mainly represented by spectroscopic technologies (UV/VIS-, IR-; dielectric-, RAMAN-spectroscopy) (55–58). In order to include process analyzers into monitoring it is not important whether measurements are performed in-line, on-line, at-line or off-line, but it is important that the data are available in time to detect deflections and to perform control actions.

After data collection, the measured raw information needs to be converted into the desired monitoring outputs. This conversion is to be performed in real-time and includes data preprocessing e.g. outlier detection, data conversion and state and parameter estimation. For these purposes, mathematical models and model-based methods can be used. Hereby measurements provide real-time data of the ongoing process, whereas the deployed model contains prior knowledge, technical and biological relationships and boundaries of the system (16). This combination of measurements and mathematical models is referred to as soft-sensor (software sensor).

In Fig. 3 the working principle of a software sensor is shown: The process states are described by x and the monitoring outputs by y. In addition to measurements, the designed inputs (u) are included as time dependent variables. The software-implemented models and estimation algorithms can hereby be of any format and structure. As a result the soft-sensor provides an estimate ($\widehat{x}$) of the current state.

Critical to the implementation of models in monitoring is the prediction and estimation ability of the model. Apart from the determination of reliable and significant model parameters (see chapter 2.1) the observability is important. An observability analysis can assess the structure of models in order to test whether the information contained in a set of measurements is sufficient for estimating model states (59). A simple approach is the numerical determination of initial values with a subset of known state trajectories, which fails in the unobservable and succeeds in the observable case. This can also be used to define the needed measurement accuracy and frequency in order to fulfil the monitoring goal. To guarantee observability the methodology can also be used to define suitable measurement combinations for specific model implementations, which has exemplarily been shown by our group for *P. pastoris* and *P. chrysogenum* processes (58).

Once the measurement scenario is defined, it needs to be interlinked with the model. Therefore, several algorithms are available, which can be summarized as observers or filters (60). The goal of the observer is to reconstruct current states of interest by real-time collected information and the given process model. Although the appropriate observer type is strongly dependent on the monitoring goal and the process model, the underlying principle is always similar. An additional model and state error *ϵ*(*t*) is added to the model representation of the previous chapter 2.1 eq. [3]). In a second relation, the so-called monitoring scenario, the monitoring outputs y with error *v*(*t*) are represented as a function of x (eq. [4]). Under the condition of observability, which means that the provided information in y is enough to reconstruct x, the current states can be estimated. Additionally, the measurement errors as well as process noise are considered as weightings (61,62).

3 $$ x(t)=f\left(t,x,\varphi, \theta \right)+\epsilon (t) $$

4 $$ y(t)=h\left(t,x,\theta \right)+v(t) $$

Using this approach, multiple measurements can be combined or unmeasured states can be reconstructed. Additionally, this methodology can be used to provide a real-time estimate based on infrequent or very noisy measurements, which can exemplarily be seen in (63). Hereby Goffaux and Wouwer (2005) implemented different observer algorithms in a cell culture process and changed measurement noise and model uncertainty. In order to cope with non-linearities and the complexity of biological systems suitable filtering algorithms need to be implemented, such as extended and unscented Kalman and particle filters (62). Kalman filters are especially suitable when the model is well-suited and only measurement and process noise occur. Particle filters allow a certain degree of model uncertainty and non-Gaussian noise distributions. In Table II examples of different monitoring implementations in biotechnology can be found.

Simple examples of successful model based monitoring are based on mass balancing (64,65,73). Thus elemental in- and out- fluxes of the reactor are measured. Considering the law of the conservation of mass, conversion rates can be determined. By applying multiple material balances, system redundancy can hereby increase the robustness of the methodology.

Kinetic models, which are more detailed and enable the description of cell internal behavior, are also well suited as soft-sensors. The limiting factor is often the system observability of complex kinetic models. Therefore, these models have to be simplified according to the monitoring goal. Aehle *et al*., for example, showed that offgas-measurement in combination with a simple model can be used to increase the reproducibility and robustness of a mammalian cell culture process (66,74).

Recent implementations by Krämer *et al*. and Golabgir *et al*. have extended the monitoring scenario by spectroscopic NIR and RAMAN measurements in order to obtain system observability of more complex models (58,70). For this purpose, the spectral data were transformed by partial least square regression (PLS) into product and substrate concentrations, which were then used as observer input. Other approaches deal with the incorporation of delayed offline measurements for real time monitoring (75–77). The additional information can help to bring the observer on the right track until the next measurement is available.

In order to provide reliable and robust monitoring as a basis for control, the inclusion of all available process information and knowledge is needed. With this regard the presented model based methods enable i) the determination of needed measurements to guarantee system observability ii) the inclusion of process knowledge in form of a model iii) possible system redundancy with multiple measurements iv) the evaluation of process and measurement noise, which finally leads to v) most probable estimates of the current state of interest.

### Process Optimization

Industrial processes aim to find process inputs (also denoted as design vector) to achieve the process goal (e.g. produce a certain product with defined specifications) and simultaneously an optimal process performance with respect to criteria like maximal profit. Additionally, those inputs have to respect physiological and technical constraints as well as product and system rationales. Optimal means getting to the best achievable results with respect to specified (might counteracting) objectives and conditions. If a reliable process model exists, it can be used to determine the optimal process inputs. In addition, the process should ideally be controlled to achieve an optimal process performance. Table III summarizes a selection of examples for model-based optimization and control from literature. In the following, typical optimization goals, variables and optimization spaces according to literature are described. Afterwards, an overview on methods and software of how to perform optimizations is presented. Finally, following a description of aspects of model based optimal control, typical challenges are presented.

**Table III:** Summary

Optimization goal	Optimization space / Constraints	Optimization variable	Optimized process / System	Algorithm	Remarks	References
Information content						
Biomass concentration, conversion of PFAP	–	media components	Synechococcus	ANNSGA (artificial neural network supported genetic algorithm)	ANN	(92)
Productivity - Offline						
Maximal biomass productivity in minimum culture time	Constraints for feed, volume, culture time	Constant/ stair case / exponential feed rate parameter	Hybridoma cell fed-batch	fminsearchcon	Offline optimization	(78)
Maximize amount of cells	Constraints for feeds and volume	Feed	Bakers yeast	Heuristic, analytical and numerical (adaptation of Jacobsons’s algorithm (93))		(94)
Productivity - Online						
Maximize productivity and yield in case of uncertainties	Volume, feed rate, operation time, amount of added substrate	Optimal feeding profile	Lysine production fed-batch	ACADO toolkit	Investigation of robust multi-objective optimal control	(91)
Process profitability (costs of product and inducer)	–	Glucose and inducer concentration	E. Coli	Pontryagin’s maximum principle	Optimal control	(95)
Maximize biohydrogen production	Constraints for feed, terminal region, culture time	Nutrient flow	Cyanobacteria fed-batch	IPOPT (after converting optimal control problem to nonlinear optimization problem with orthogonal collocation)	Simulation MPC with parameter estimation	(79)
Maximize Productivity	Max volume	Feed	Steptomyces tendae		MPC	(80)
Robustness						
Control glucose to a setpoint	–	Glucose feed rate profile	CHO fed-batch	SQP (sequential quadratic programming)	MPC	(81)
Control consumed oxygen to a setpoint	–	Glutamine feed rate	CHO fed-batch	Simplex	MPC	(74)

Mathematically, optimization problems are typically interpreted as minimization problems of an objective function. In general, three types of optimization objectives typically arising in different stages of the process lifecycle can be distinguished. These are optimizing (i) information content, (ii) productivity and (iii) robustness and reproducibility: (i) Especially but not only during process development optimization algorithms are used to find the parameters of a process model by minimizing the model deviation from the given data (see chapter 2.1.1) or to maximize the information content of planned experiments (see chapter 2.1.2) to obtain adequate process models. (ii) When having a reliable process model, the optimization of the productivity of the process is typically aimed at, e.g. to achieve highest amounts of biomass or product at the end of the process (78–80). (iii) Finally, robustness and reproducibility of an optimized process are typical goals. In this case the objective is usually a minimal deviation from identified (optimal) set points during the whole process. Examples are dissolved oxygen or pH, but also variables like metabolite concentration (81), growth rate or a process variable related to it like the oxygen consumption rate (74). In these cases a dynamic model is needed (see chapter 2.1.1).

A fact to be considered during model development is that only inputs that are included in the process model can be optimized (see chapter 2.1.1). For bioprocesses those are usually feed-rates or initial values. The optimization space is frequently constrained, as shown in Fig. 4: on the one hand, physiological and technical constraints like maximal volume, feed rates or culture time (78,79) have to be considered - on the other hand, the optimization space has to be restricted to an area where the model can be trusted, a region the exact location of which is typically hard to define (see chapter 2.1.1). Because product quality is the priority aim of pharmaceutical production processes, the design space is limited by certain product rationales (e.g. pH and temperature area) too. In addition to that, reducing the size of the optimization space also can speed up the computation time which is needed for time-sensitive optimization tasks. The optimization space is strongly dependent on the process lifecycle. New models, monitoring methods, control strategies, regulatory requirements and changed costs can lead to an expansion of the optimization space and therefore to new optimal design vectors.

There are various methods to solve optimization problems. In some cases the optimization problem can be solved analytically, which means a solution function (for example in integral form) can be obtained. However, frequently (nonlinear) numerical algorithms have to be applied. Various optimization algorithms exist, detailed descriptions can be found in textbooks like (85) or the review of (86). For bioprocesses, frequent implementations of the Nelder-Mead simplex algorithm (fminsearch and its derivates) (87) or differential evolution (88) in MATLAB are used (74,78,83). Another powerful method for large-scale nonlinear optimization is the software package IPOPT (89) e.g. used by (79) for optimizing biohydrogen production. More applied algorithms are listed in Table III. When choosing the optimization algorithm, one has to ponder aspects like the number of variables to be optimized, the complexity of the model, the implementation environment or the acceptable duration of the optimization. The last point is of major importance when performing optimizations during the process. In case the optimal design vector is time-dependent it might has to be parametrized. This is frequently done by discretizing the input signal via partially constant, linear or parabolic functions (also termed as zero, first or second order hold). Simulations are a valuable tool to investigate configuration details e.g. how to parameterize the design vector. This can also help to ensure a fast computation (80).

When the optimal values of the process inputs are found, various possibilities for controlling the process to achieve the desired optimal performance exist: a simple method is to determine the optimal design vector once and control the process on those predefined set points. This approach is state of the art in most production processes.

However, this control method possibly fails when process deviations occur due to model uncertainties or unknown or neglected process disturbances which are not considered previously. The reason is that this strategy does not consider the real values of the process outputs (the controlled variables) during manipulating the inputs (the manipulated variables). This can lead to unwanted process behavior: (80) computed optimal profiles for three feeds (ammonium, phosphate, glucose) based on a mechanistic model. They studied the effect of model uncertainties by varying the model parameters and applying those feed profiles determined with the initial parameters. The results revealed a high dependency of end product (the optimization goal) on the model parameters: in 60% of the simulations less product than in the original case was achieved. They concluded that this can be avoided by applying closed-loop control. In this case the manipulated variables are adjusted based on the values of the controlled variables. Besides classic closed-loop controllers like PID controllers, a well-known and powerful representative method is model predictive control (MPC) (80–85): a dynamic model is used to find the optimal inputs with respect to a defined objective function as described above. However, instead of performing the computation only once in the beginning, the optimization is repeated after a defined control horizon to react towards process deviations. Therefore, the optimization problem has to be solved in real-time, which demands robust and fast optimization algorithms. In order to be able to discover process deviations information about the current process state is needed. Depending on the measurement environment monitoring strategies as described in chapter 2.2 have to be applied.

Dynamic optimization of bioprocesses is linked with several challenges. E.g., in case of multiple objectives it is difficult to choose an optimal solution: typically, there can be counter-acting objectives in such that one objective can only be improved by worsening the other, which implies a trade-off is needed. This set of solutions is known as Pareto front. More theory on this topic can e.g. be found in the textbook by (90). Another aspect is robustness towards process deviations and model uncertainties. One way to deal with this is presented by (91), who investigated robust multi-objective optimal control in case of model uncertainties by interpreting robustness as additional objective. Another typically occurring phenomenon is, that the optimal design vector lies at the boundaries of the optimization space. One the one hand, this can be critical if the optimization space is not defined properly, for example due to limited knowledge about the validity space of the model. On the other hand this implies that the process might be optimized by increasing the optimization space e.g. by deriving more knowledge to increase the model validity space and improve the model or by technical innovations.

Summing up, optimization tasks occur during different stages of the process lifecycle, with the highly diverse goals of maximal information content, productivity and robustness and reproducibility, respectively. Methods for optimization and control are limited by the quality and the inputs of the model. In addition to that, closed loop optimal control is also limited by issues like process noise or uncertainties of the model and the system. Therefore a suitable monitoring strategy has to be established and suitable observers have to be applied. In addition to that, if optimization has to be performed online and probably unsupervised, fast and trustworthy algorithms are demanded. However, in those cases, where this is fulfilled, MPC is a valuable tool to achieve optimal processes.

### Data Mining for Detection of Disturbance Variables

Although sophisticated control strategies are applied to modern processes (achieved using the above described methods with respect to determination, monitoring and optimization of CPPs), fluctuations in process performance inevitably occur. For that reason, continuous process improvement is necessary, which can be achieved by data mining techniques in order to detect disturbance variables.

Generally, every bioprocess includes known but neglected or tolerated disturbances, such as the control ranges of process parameters like pH, dissolved oxygen, feeding profiles etc. On the other side, there are unknown disturbances that might undermine process robustness and that should be identified in later stages of the process development or during process improvement. In the following, we want to focus on the upstream of biopharmaceutical processes as this is the major source of disturbances. According to the exemplification of the bioreactor as a disperse multiphase-system, these unknown disturbances can be grouped in the following classes as follows:

1. Biomass as disturbance variable, either due the genotype (e.g. repression or induction of certain genes) or phenotype (e.g. morphological changes)
2. The composition of or single substances in the fluid phases as disturbance variable (e.g. raw material variability, metabolites, process additives)
3. Physical and local characteristics such as inhomogeneities as disturbance variable (e.g. improper dispersal of base/ acid or feeds, inhomogeneities in dissolved oxygen etc.)

The detection of disturbance variables aims at enhancing the understanding of process fluctuations, thereby increasing process robustness or process performance and can finally even lead to improvement of control strategies (see chapters 1 and 2.3). The ability of process intervention according to knowledge gained via an analysis of disturbance variables is strongly coupled to the optimization and especially to the innovation space. This means that a possible intervention is limited by the biological system itself, e.g. physiological parameters like maximal specific uptake rates, but also by external factors such as technical feasibility or logistical and organizational factors, e.g. time line for upstream to downstream processing, shift work etc. One the one hand, the development or implementation of new analytical methods or probes for the characterization of the system and its disturbances can lead to an extension of the innovation space of the investigated bioprocess and thereby enhance process control strategies. On the other hand, technical or organizational constraints can restrict process intervention – within the borders of the innovation space - although a disturbance was successfully detected (Fig. 4).

Generally, the detection of important disturbance variables follows a data-driven knowledge discovery approach, mainly focusing on data mining methods (Fig. 5), i.e. statistical methods to extract information from large data sets. Risk assessment tools are commonly used for process development (2) and can also facilitate the identification of possible disturbance classes (see definition above) within the design space of the process. A prominent example of these tools is the Ishikawa (or fishbone) diagram, which illustrates that this form of process improvement is done at later stages of the process development as some prior knowledge about the process is necessary (i.e. QbD approach). According to the outcome of the risk assessment, data has to be generated or compiled. Every modern biotechnology production plant is equipped with systems that record and archive continuous and intermittent data of every process. These historical data can be used for data mining and the identification of disturbance variables - even including known but neglected disturbances. Examples of the assessment of historical data are given in (96–98). (99), for instance, used a three-step approach previously introduced by (100,101) in order to optimize an *E.coli* process for green fluorescent protein production.

Often, historical data do not represent the probable disturbance class well enough, which is why additional data are needed. These data are commonly generated via analytical measurements of specific components (e.g. HPLC, IC or ICP analysis), for instance of the raw material for media production. Examples of this approach are given by (102) and by our group (103), who focused on the detailed characterization of complex raw material. As this approach is very laborious and analytically challenging fingerprinting methods such as near infrared (NIR), mid infrared (MIR) or (2D)-fluorescence spectroscopy can be applied to complex matrices. These methods generate an overall but still specific description of the composition of a complex material or media (e.g. a spectra) without the identification of certain substances, i.e. a fingerprint of the material. Spectroscopic fingerprinting methods were applied by (104–108) in order to determine the variability and disturbances of applied raw material.

Before data mining techniques can be applied, it should be noted that the characteristics of bioprocess data is its heterogeneity with respect to time scale. As already mentioned in chapter 2.2, bioprocess data can be continuous measurements, intermittent measurements or even one-time measurements at the beginning or the end of the process, such as raw material attributes or process titer, respectively. Hence, before data analysis can be started, preprocessing techniques, feature selection or even dimensionality reduction has to be performed. Examples of these techniques applied for historical datasets are described in (96), such as filter and wrapper methods or principle component analysis (PCA) for dimensionality reduction. If additional analytical data are generated at one point of time, e.g. measurements of specific components or fingerprinting data of the used raw material, other preprocessing methods have to be applied. For fingerprinting spectra, first, second or third order derivatives are commonly used in order to reduce noise from the spectral data. Additionally, data can be mean-centered or normalized, depending on the statistical method that is used for further analysis (109–113). In the following step the actual data mining starts, which can be categorized in descriptive or predictive approaches (96) (Table IV).

**Table IV:** Various Methods are Available for the Data to Information Approach, which is applied for the Identification and Minimization of Disturbance Variables. The Most Common Ones are Stated here Including Information about Linearity, Advantages and Disadvantages as well as References to Literature

Approach	Method	Advantages	Disadvantages	Output	Method literature	Application literature
Descriptive	PCA	• Orthogonal • Dimensionality reduction • Easily applicable • Provides overview of input matrix • Classification of data	• Difficult to interpret if more PCs are significant • no correlations with process response possible • linear	• Loadings ➔ describes the correlation between variables in an orthogonal manner • Scores ➔ shows grouping/ clustering/ patterns/ trends ➔ facilitates interpretation due to additional dimensionality reduction	(120)	(102,103,107,108,121–123)
Descriptive	Cluster analysis (CA)	• Classification of data • Multiple algorithms are available ➔ adaption to problem statement possible	• No dimensionality reduction ➔ complicates the identification of trends • Linear • No Correlation with process response	• Dendrogramm ➔ clusters can be seen and especially the distance between clusters can be analyzed		(98,121,122)
Descriptive and predictive	PLS-DA	• Dimensionality reduction • Prediction of group membership • Classification of data • Easily applicable	• Linear • Y-variable (i.e. class) has to be declared before analysis • Knowledge about method necessary (choice of threshold, PLS1 or PLS2) • Overfitting	• Scores ➔ shows grouping/ clustering/ patterns/ trends ➔ facilitates interpretation due to additional dimensionality reduction • Weights/ loadings ➔ relates classifier to underlying variable	(115,116)	
Descriptive and predictive	OPLS-DA	• Orthogonal • see PLS-DA	• see PLS-DA	• see PLS-DA		(104,117)
Predictive	MLR	• Easily applicable • Correlation with process response	• not applicable for fingerprinting analysis (due to collinearities) • linear	• ANOVA validation • Coefficients with confidence intervals ➔ representing variables that correlate with process response		(124)
Predictive	PLS	• Dimensionality reduction • Correlation with process response • Variable ranking available • Easily applicable	• Not orthogonal • Correlations are assumed to be linear (only “quasi-nonlinear” algorithmic adaptations • available like Poly-PLS or Spline-PLS) • Small validity space • linear	• Observed *vs* predicted • Coefficients with confidence intervals ➔ representing variables that correlate with process response	(119,125,126)	(108,121,123,124)
Predictive	PCR	• Dimensionality reduction • Easily applicable • Orthogonal • Correlation with process response	• Difficult to interpret if more PCs are significant • Correlations are assumed to be linear	• see PCA and MLR	(127)	(128,129)
Predictive	ANN	• Correlation with process response • Adaptive learning • Self-organization • Fault tolerance via redundant coding • Real-time operating ability • Easy insertion into existing technologies • non linear	• Mathematically demanding • difficult to implement for process development • iterative workflow • dependence of final result on initial parameters • tendency to overfitting • high training time and computational resources • non-uniqueness of final result	• Observed *vs* predicted (cross validation)	(119,130)	(124)
Predictive	SVM	• see ANN • handling high dimensional input vectors	• see ANN	• see ANN	(119)	

In the descriptive approach methods for discriminant analysis are applied in order to identify patterns or clusters in the dataset. Common methods are PCA, e.g. applied by (102,103) and cluster analysis (98). For the predictive approach methods are applied that allow correlation analysis, i.e. the preprocessed data or selected features are correlated with process outcomes (i.e. CQAs and productivity) in order to identify possible relationships. Typical methods are multiple linear regression (MLR), partial least squares (PLS) regression and artificial neural networks (ANN). There are also modifications of these methods available that overcome certain drawbacks of the original method as well as relatively new methods such as support vector machines (SVM).

Jose *et al*. analyzed two raw materials via two fingerprinting techniques (105). In order to combine the spectra of these two materials PCA models for both raw materials were generated and the scores of these models were used for the generation of an interval partial least squares (iPLS) regression model which allowed a correlation between raw material quality and product yield and titer. iPLS is a graphical extension of regular PLS models. It divides spectral data into equidistant subintervals of which validated calibration models are developed. Hence, this method allows to depict relevant information in different spectral subdivisions and is able to remove interferences from other regions (114). Another method proposed by Gao *et al*. for the identification of raw material and process performance is the orthogonal partial least squares – discriminant analysis (OPLS-DA) (104). This method equals partial least squares – discriminant analysis (PLS-DA) which is a combination of canonical correlation analysis and linear discriminant analysis Thus, providing descriptive as well as predictive information (115,116). The integration of an orthogonal signal correction (OSC)-filter, which should allow the separation between predictive and non-predictive variation, should improve the interpretation of the model (117,118). Nevertheless, the superiority of OPLS-DA over PLS-DA is critically discussed among experts. Balabin *et al*. introduced an extension of ANN, namely support vector machines (SVM), for spectroscopic calibration and as data mining technique (119). It has the advantage of providing global models that are often unique, which is a benefit compared to normal ANN.

Descriptive as well as predictive methods result in the generation of hypotheses about disturbances, crucial parameters or interactions. These hypotheses have to be evaluated or experimentally verified by experts (e.g. via experimental design as mentioned in chapter 2.1.2) before they can be implemented in the control strategy. At this stage the control loop (Fig. 1) can be restarted by the integration of gained knowledge in the model or even by the introduction of new CPPs or kPPs. This approach can additionally result in the improvement of product quality and productivity.

In general there are three major challenges in process improvement via detection of disturbance variables: The identification of an adequate analytical method for in-depth investigation of disturbance variables, such as cell morphology, raw material or scale-up effects (e.g. inhomogeneties, biomass segregation), is demanding, especially with increasing complexity of the process. The knowledge about method errors and general deviations during the process is necessary in order to allow adequate conclusions from data mining. Additionally, the choice of the appropriate statistical method that is applied to the data compilation is crucial to achieving meaningful patterns, clusters and correlations and has also an impact on the interpretability of the results.

Summing up, for continuous process improvement, the evaluation of both historical data as well as the generation of new data with respect to probable disturbance variables is necessary. Data mining of these huge datasets allows the generation of hypotheses which can be verified by experiments. Gained knowledge can further on be implemented in existing models in order to improve process robustness and performance (Fig. 2).

## Conclusions

During the biopharmaceutical process lifecycle, countless challenges arise: uncontrollable external conditions, fluctuations in raw material, inaccuracies in process control and continuous innovations - and they all affect the process performance over time. The trend of the last few years has clearly pointed towards a model approach in order to ensure knowledge transfer during the entire process lifecycle and, additionally, during different processes. Model-based methods allow the applicability of the stored knowledge. In the presented review the applicability of model-based methods in order to ensure control has been shown. To reach the goal of control four challenges were investigated: I) generation of process knowledge, II) process monitoring, III) process optimization and IV) continuous improvement of the process (Fig. 1).

The first challenge includes the identification of CPPs and kPPs, hence, the generation of process knowledge. If relations and interactions within the process are understood, the main challenge is the setup and the verification of process models in order to predict a target value (CPP, kPP or CQA). This is a critical step because the model quality has an impact on accuracy, precision, applicability and the validity area of all model-based methods. Main issues in the field of modelling are a lack of experts and tools for the model setup in biopharmaceutical production processes. In addition, process-models should be extended or adapted during the whole process lifecycle. Therefore, modelling is a typical bottleneck for the application of model-based methods in industrial processes. In order to overcome this problem, we presented modelling workflows for the setup of models. Additionally, methods for the generation of information during experiments by model-based experimental design are presented.

The second challenge is an adequate process monitoring. The combination of real-time measurements and model-based methods like observers allow an optimal usage of monitoring capacities. Model-based methods are already widespread and accepted in the area of process monitoring since they allow the estimation of hard or not measureable parameters and variables, which are necessary for subsequent control tasks. The bottleneck of monitoring methods is mainly the transferability between different processes and scales concerning measurement methods and software environment. During the process lifecycle new real-time measurement sensors, changing process models and new control tasks should be considered in the process monitoring concept.

The third challenge is process optimization and process control. First of all, a proper definition of the optimization objective is needed. Especially in case of multiple objectives an adequate weighting of the different goals is not easy but important. The second task is to find an optimal design vector for the process. Model-based methods are valuable tools to declare the optimum. Nevertheless, multidimensional optimization tasks are generally hard to implement as well as computationally demanding. Furthermore, successful optimization highly depends on the model quality as well as knowledge about the validity space of the model.

The fourth challenge is the continuous improvement of the process based on additional research and historical data assessment. Therefore, datamining tools are widespread and accepted as model-based methods in order to generate hypotheses, which can be experimentally evaluated and furthermore gained knowledge can be included in the process model. Bottleneck of these datamining tools are mainly the availability of adequate measurement methods for the generation of additional data and the interpretability of descriptive as well as predictive model-based methods.

Irrespective of the availability of model-based methods, a certain acceptance of these methods in the biotechnological community has to be generated. Hence, the benefits of the application of model-based methods on process development and production have to be demonstrated. Additionally, the training of the users is of great importance as well as the presentation of all methods in more user-friendly tools. In combination with continuous support and further development of the process model, model-based methods are powerful tools to ensure the overall goal of biopharmaceutical processes, i.e. the guarantee of high product quality.

## Abbreviations

ANN: Artificial neural networks
CHO: Chinese hamster ovary
CMA: Critical material attributes
CMB-DoE: Continuous model-based experimental design
CPP: Critical process parameters
CQA: Critical quality attributes
DoE: Design of experiments
E.coli: *Escherichia coli*
HPLC: High performance liquid chromatography
IC: Ion chromatography
ICH: International Conference on Harmonization
ICP: Induced coupled plasma
iPLS: Interval partial least squares
IR: Infrared
kPP: Key process parameters
M^3^C: Measurement, monitoring, modeling and control
MB-DoE: Model-based design of experiments
MIR: Mid-infrared
MLR: Multiple linear regression
MPC: Model predictive control
NIR: Near-infrared
NRMSE: Normalized root mean square error
OPLS-DA: Orthogonal partial least squares-discriminate analysis
OSC: Orthogonal signal correction
PAT: Process analytical technology
PCA: Principle component analysis
PLS: Partial least squares
PLS-DA: Partial least squares-discriminate analysis
QbD: Quality by design
QTPP: Quality target product profile
SQP: Sequential quadratic programming
SSE: Sum of square errors
SVM: Supported vector machine
